# Mapping Lean Six Sigma in Healthcare: A Bibliometric Analysis Revealing Research Gaps in Developing Countries

**DOI:** 10.7759/cureus.88692

**Published:** 2025-07-24

**Authors:** Seep Sonali, Shweta Shenoy

**Affiliations:** 1 MYAS-GNDU Department of Sports Sciences and Medicine, Guru Nanak Dev University, Amritsar, IND

**Keywords:** critical, factors, healthcare, indian, lean six sigma, success

## Abstract

Lean Six Sigma (LSS) is a process improvement methodology that combines Lean’s focus on eliminating non-value-adding activities with Six Sigma’s data-driven approach to reduce defects and enhance efficiency and quality. Globally, the healthcare sector faces challenges, such as resource constraints, rising patient demands, and infrastructural limitations, with these challenges particularly pronounced in developing countries, like India, making it a promising tool for optimizing processes and improving patient outcomes. Despite its potential, the adoption of LSS in Indian healthcare is limited, and critical success factors (CSFs) such as leadership, communication, training, and organizational culture remain underexplored. This study investigated global research trends, thematic interconnections, and identified geographical gaps in LSS implementation, particularly revealing the underrepresentation of developing countries like India in the literature. A bibliometric analysis was conducted using data from the Scopus, PubMed, and Web of Science databases, covering peer-reviewed English-language articles published between January 2012 and April 2024, followed by a qualitative systematic review of the identified studies (n = 8). A targeted search strategy employed keywords, such as “Lean Six Sigma,” “Critical Success Factors,” “Service Quality,” AND, OR “Healthcare,” combined with Boolean operators (AND, OR). The inclusion criteria required articles to address service quality, focusing on CSFs involved in the successful implementation of LSS in healthcare settings. The exclusion criteria excluded non-peer-reviewed studies, conference abstracts, articles not in the English language, and non-healthcare studies. From the 262 retrieved articles, metadata, including titles, authors, abstracts, and keywords, were extracted, cleaned for duplicates, and analyzed using VOSviewer, version 1.6.20 (Leiden University) to map keyword co-occurrences and co-authorship networks. The analysis identified a robust global LSS research ecosystem, with core terms such as “Service Quality”, “Lean”, and “Six Sigma, forming a dense central cluster, red coloured, linked to healthcare themes such as “CSFs” (68 co-occurrences) and “patient safety” (44 co-occurrences). The analysis revealed significant geographical disparities, with developing countries underrepresented; for instance, India contributed only 1 out of 262 (0.38%) publications, highlighting a critical research gap in emerging economies. Organizational Culture (39 co-occurrences) and Training (35 co-occurrences) were universal CSFs in healthcare studies, followed by Communication (28 co-occurrences) as per the bibliometric keyword analysis. Whereas a qualitative systematic review revealed Leadership and management as the most universal factors (8/8 studies). Communication was prominent too (6/8), followed by training and expertise, and organizational readiness (5/8 and 3/8 each), whereas technology integration and employee retention, and culture were least cited (1/8). Western nations dominate the research output, highlighting geographical disparities. The neglect of technology integration, employee retention, and data-driven decision making as CSFs underscores barriers in resource-constrained environments. Future research should prioritize India-specific studies and integrate cost-effective solutions and longitudinal assessments to enhance LSS adoption and improve health care delivery.

## Introduction and background

Lean Six Sigma (LSS) is a powerful process improvement methodology that combines Lean’s focus on eliminating non-value-adding activities with Six Sigma’s rigorous data-driven approach to reduce process variation and defects [1]. Lean, rooted in Toyota’s production system, aims to streamline processes by removing waste, such as overproduction, waiting times, and unnecessary inventory; Six Sigma, pioneered by Motorola, employs statistical tools to achieve near-perfect quality through defect reduction [2]. LSS fosters operational excellence by enhancing efficiency, reducing costs, and improving quality, making it a versatile framework for both manufacturing and service sectors [3]. In healthcare, LSS has gained prominence in addressing challenges such as long patient wait times, medical errors, and resource inefficiencies, ultimately improving patient outcomes and organizational performance [1,4]. The foundational tenets of Six Sigma and Lean exhibit numerous parallels that account for their concurrent implementation in practice. Ultimately, both methodologies yield comparable value propositions for customers and organizations [5].

In India, the healthcare sector presents a unique landscape characterized by significant heterogeneity, with resource-constrained public hospitals coexisting alongside sophisticated private facilities [6]. The sector faces mounting pressure to deliver high-quality, affordable care amid rising patient expectations, limited budgets, and infrastructural challenges [3]. LSS offers a promising solution by enabling hospitals to optimize processes, enhance service delivery, and achieve sustainable improvements [6]. LSS transcends mere methodology or the possession of requisite tools for enhancement; it embodies a mindset and psychological framework essential for effectuating change. Given that the healthcare sector is characterized by its focus on human resources and process orientation, it presents an ideal context for the application of the Lean and Six-Sigma principles.

However, the successful implementation of LSS in healthcare depends on critical success factors (CSFs), key elements that must be effectively managed to ensure project success [6-8]. These CSFs, such as leadership commitment, comprehensive training, robust communication, and a supportive organizational structure, are well documented in the global literature, but remain underexplored in the Indian healthcare context [6,7]. This research gap is critical, as India’s socio-economic, cultural, and operational dynamics necessitate tailored strategies for LSS adoption.

Nevertheless, the healthcare industry remains largely unaware of the sustainable advantages associated with LSS methodology, particularly in developing countries such as India. Consequently, the objective of this review was to perform a bibliometric analysis aimed at investigating the research trajectories, trends, and interconnections between LSS and CSFs within the healthcare domain, with an emphasis on pinpointing the research deficiencies present in Indian healthcare environments. Through an examination of literature sourced from various databases, this review delineated keyword co-occurrences, illuminated research activity clusters, and emphasized the inadequate exploration of LSS-CSFs in the context of Indian healthcare relative to other domains such as manufacturing, thereby substantiating the necessity for additional inquiry in this area.

## Review

The methodology for the bibliometric analysis was structured to systematically explore research trends, patterns, and relationships in LSS and CSFs within the healthcare sector, with a specific focus on identifying research gaps in Indian healthcare settings.

Data collection

Bibliometric analysis began with data collection from three prominent academic databases: Scopus, PubMed, and the Web of Science. These databases were selected because of their extensive coverage of peer-reviewed literature in the healthcare, management, and related fields, ensuring a reliable and diverse dataset. A targeted search strategy was employed using keywords such as “Lean Six Sigma,” “Service Quality,” “Critical Success Factors,” AND, OR “Healthcare,” combined with Boolean operators (AND, OR) to focus on LSS implementation studies. The search strategy was designed to capture global research trends without geographical restrictions, allowing for comprehensive mapping of worldwide LSS implementation in healthcare. To maintain analytical consistency, the search was limited to articles published in English.

The inclusion criteria required articles to explicitly address LSS, CSFs, or service quality in healthcare settings, while the exclusion criteria eliminated non-peer-reviewed works, conference abstracts, and studies focused solely on non-healthcare sectors, such as manufacturing. The timeframe was specified as January 2012 to April 2024, as indicated by the Visualization of Similarities viewer (VOSviewer, version 1.6.20, Centre for Science and Technology Studies, Leiden University, Leiden, Netherlands).

Data extraction and cleaning

**Figure 1:**
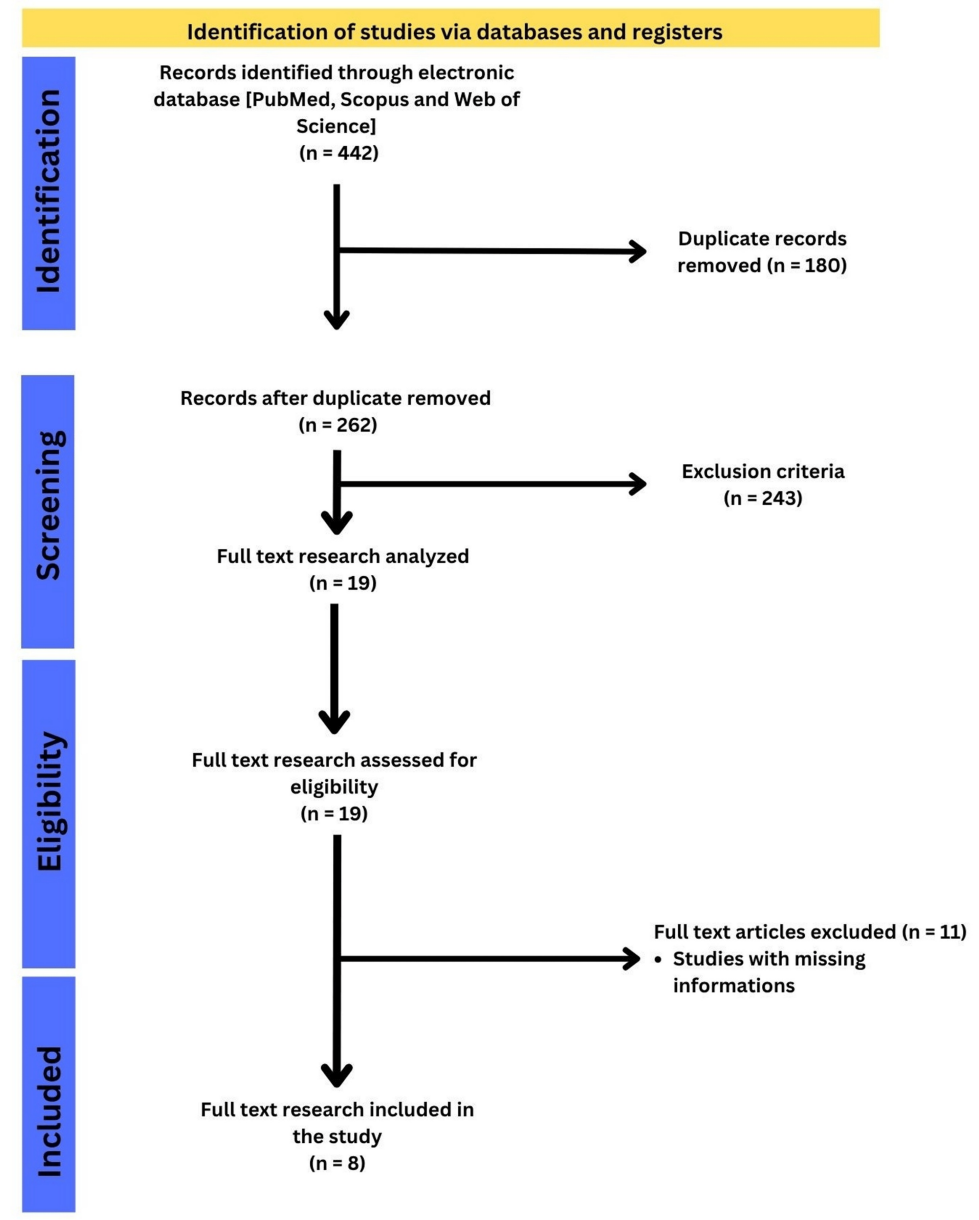
Preferred Reporting Items for Systematic Reviews and Meta-Analyses (PRISMA) flowchart for the review.

From the database searches, 262 articles were initially retrieved and exported in compatible formats (e.g., Comma-Separated Values or Research Information Systems) for bibliometric processing. The systematic review involved steps like the identification process, screening process, eligibility criteria, and the final selection of articles. Metadata extracted included article titles, authors, publication years, journals, abstracts, and keywords to enable comprehensive analysis. A rigorous cleaning process was conducted to remove duplicates and irrelevant entries and to ensure the accuracy of the dataset. This process is depicted in the flow diagram (Figure 1), which details the refinement process. While the broader systematic literature review was narrowed down to eight healthcare-specific studies, the bibliometric analysis retained a larger dataset to capture global and Indian trends in CSFs and LSS to improve service quality, focusing on healthcare-specific patterns. 

Bibliometric analysis tool and parameters

The analysis was conducted using VOSviewer, version 1.6.20 (Centre for Science and Technology Studies, Leiden University), a widely used tool for visualizing bibliometric networks, particularly for mapping keyword co-occurrences, co-authorship, and citation patterns. The focus was on keyword co-occurrence analysis to identify the research clusters and thematic relationships. A minimum threshold was set, typically requiring keywords to appear in at least five articles to ensure meaningful connections and avoid noise in the visualization. Nodes in the VOSviewer output represented keywords, with edges indicating the strength of co-occurrence relationships. This setup allowed the identification of dominant themes and gaps in the literature, particularly in the healthcare sector.

Visualization and interpretation of results

VOSviewer outputs provide visual representations of keyword co-occurrence. These visualizations were analyzed to identify dominant research themes, emerging areas, and gaps, particularly the limited exploration of LSS-CSFs in the healthcare sector globally, particularly the limited exploration of LSS-CSFs in Indian healthcare, which justified the study’s focus. The bibliometric maps also quantified the relative research output in healthcare compared to other sectors, providing a clear rationale for further investigation.

Validation of findings

To ensure the reliability of the bibliometric results, the findings were cross-referenced with existing literature reviews, such as Albliwi et al. [3], which similarly noted gaps in LSS research within the service sectors. The identified clusters were compared to the findings of a broader systematic literature review to ensure alignment with the overall research objectives. This cross-validation strengthened the credibility of the bibliometric analysis and its role in highlighting the under-researched Indian health care context.

Results

**Figure 2:**
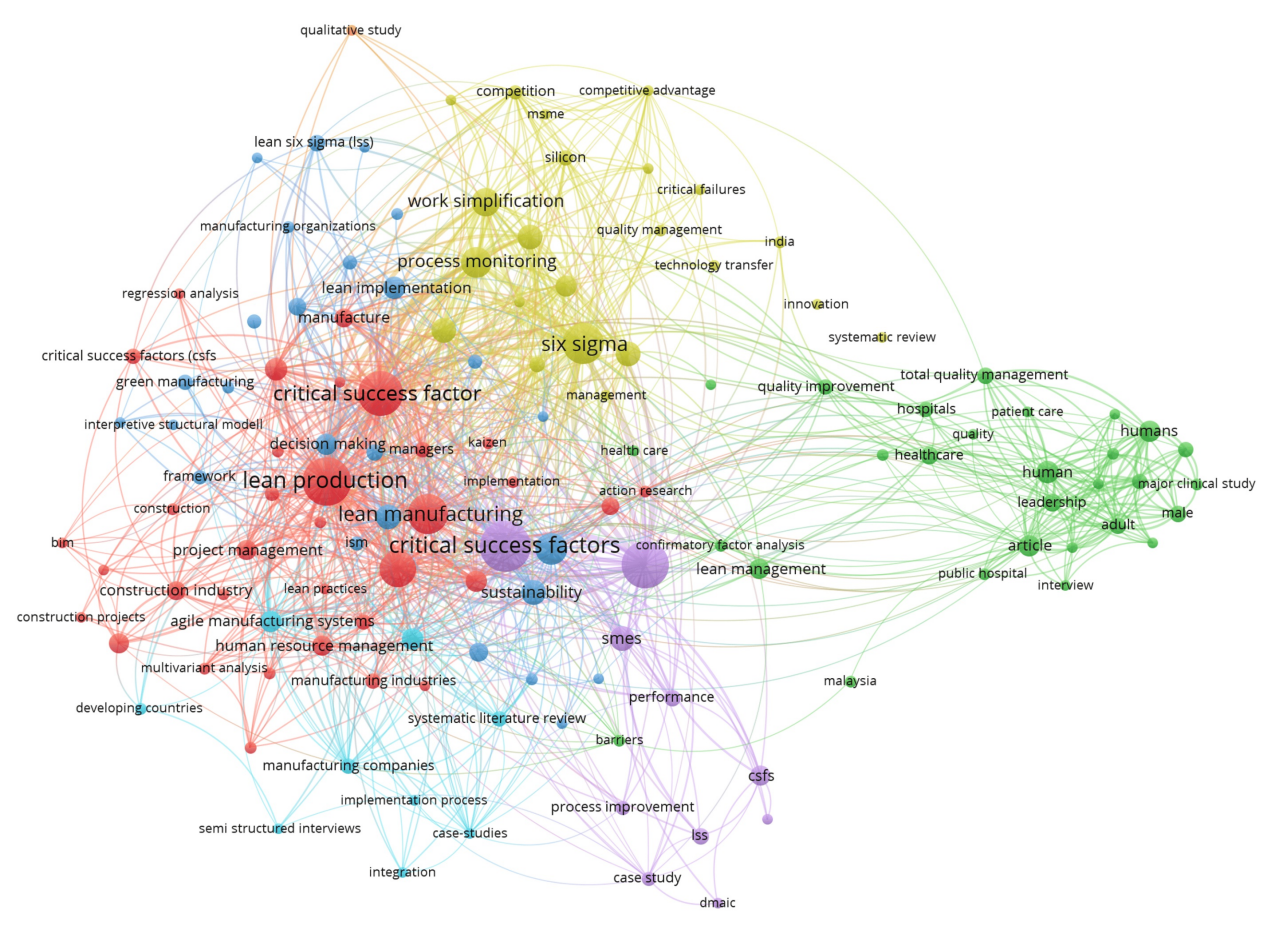
Keyword co-occurrence network visualization of Lean Six Sigma (LSS) research generated using Visualization of Similarities viewer (VOSviewer) using Scopus database. This map displays clusters of frequently co-occurring keywords extracted from bibliographic data. Each node represents a keyword, with the size indicating the frequency of occurrence. Lines between nodes represent co-occurrence links, and the distance between nodes reflects the relatedness of terms. Different colors represent thematic clusters identified by the software's clustering algorithm. The red cluster represented core methodological concepts central to LSS. The green cluster highlighted key application domains, while the blue cluster emphasized performance outcomes. The yellow cluster captured related improvement strategies and integration with other frameworks, and the purple cluster focused on statistical tools and techniques.

Bibliometric co-occurrence analysis using VOS-viewer generated a keyword network map that revealed the thematic structure of this research. The visualization identified multiple clusters, each representing a distinct subdomain. Core methodological terms such as "Six Sigma," "Lean," and "CSFs" formed a central and dense cluster, indicating their foundational role across the literature. Surrounding clusters included application-focused terms like "healthcare," "manufacturing," “quality improvement,” and "process improvement," highlighting the interdisciplinary and practical nature of the CSFs involved in LSS implementation. Other peripheral clusters addressed performance indicators such as "cost reduction," "efficiency," and "quality control”. The interconnectedness of clusters suggests a high degree of integration between methodology, application, and outcomes within the LSS research landscape (Figure 2).

**Figure 3:**
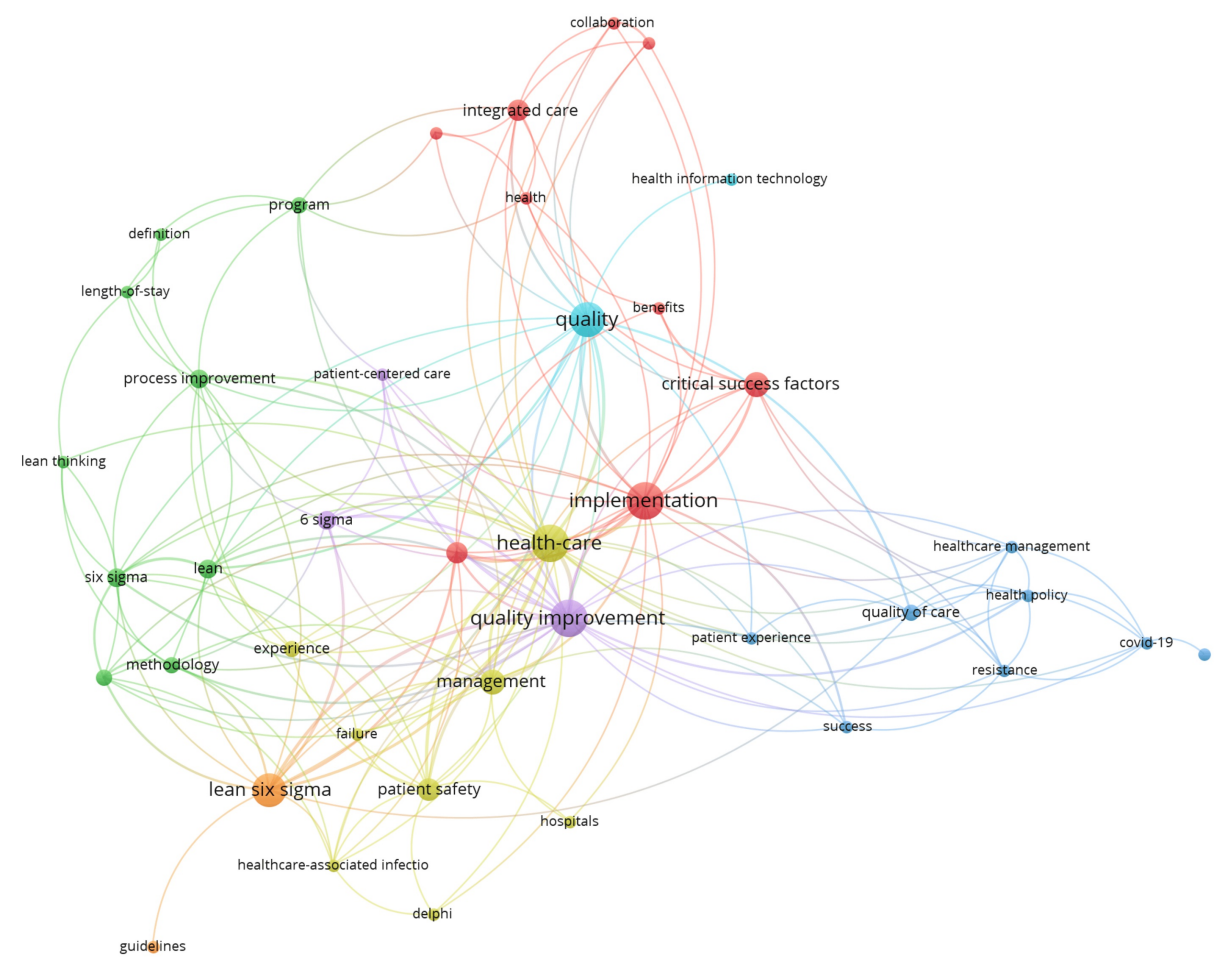
Author co-authorship network visualization in Lean Six Sigma (LSS) research generated using Visualization of Similarities viewer (VOSviewer) using Web of Science database. VOSviewer results of co-ocurrences in papers published in high-quality journals indexed in Web of Science: Display details show weaker linkages in all the colored networks for Lean thinking, LSS, and critical success factors, which signifies a research gap and the relevance to explore this topic. The green cluster suggests an emerging concept contributing to a niche area within the field.

Co-authorship network visualization highlighted that organizational culture, training, and data-driven decision-making frequently emphasized CSFs in LSS research. Prominent authors, such as Laureani and Antony [8] and Al-Balushi et al. [4], appeared as central nodes within major clusters, indicating their significant collaborative contributions and influence in the field. The clustered structure of the map reflected distinct thematic concentrations, with some focusing on practical applications through case studies, while others emphasized empirical validation via surveys. Despite these strengths, the network also revealed gaps in theoretical development and limited exploration of rural healthcare settings. Additionally, the density and expansion of clusters post-2012 suggested a notable increase in CSF's impact on LSS research activity, mirroring global trends toward quality and process improvement in diverse sectors (Figure 3).

The bibliometric analysis revealed key research trends in 262 publications. LSS (92 co-occurrences, red cluster) and service quality (74, red cluster) dominate, indicating strong integration in quality and process improvement studies. CSFs (68, blue) and organizational culture (39, blue) formed distinct thematic clusters, highlighting managerial focus. Healthcare (61, green) and training (35, green) were linked to operational implementation, whereas patient safety (44, yellow) and communication (28, yellow) emphasized outcomes. India (8 co-occurrences) appeared in the keyword network, but its limited presence compared to other geographical terms highlights the research gap in this major developing economy. VOSviewer mapping confirmed the LSS centrality with interconnected themes in quality management, healthcare efficiency, and organizational drivers, reflecting a multidisciplinary yet structured research landscape (Table 1).

**Table 1:** Based on initial search results (n = 262). VOSviewer: Visualization of Similarities viewer.

Keyword	Co-occurrence count	Cluster color (as per VOSviewer)
Lean Six Sigma	92	Red
Service quality	74	Red
Critical success factor	68	Blue
Healthcare	61	Green
Patient safety	44	Yellow
Organizational culture	39	Blue
Training	35	Green
Communication	28	Yellow
India	8	Green

Bibliometric analysis identified the top ten most-cited articles in LSS-CSF healthcare research [9-15]. Laureani and Antony [8] led to 150+ citations, followed by Al-Balushi et al. [4] led to 120+ citations, and Noori [9] led to 100+ citations. Kumar et al. [10] and Swarnakar et al. [7] ranked next, cited 80+ and 65+ times, respectively. Ali and Alolayyan [11] received 60+ citations, while Spagnol et al. [12] led to 55+ citations. Sidek and Martins [13], Rathi et al. [14], and Naidoo and Fields [15] rounded out the list with 50+, 40+ citations, and 38+ citations, respectively, reflecting the key contributions to LSS in the field of healthcare quality management (Table 2).

**Table 2:** Academic weight and citation trends based on initial search results (n = 262).

Rank	Author(s)	Year	Title	Journal	Citations
1	Laureani and Antony [8]	2018	Leadership - a critical success factor for the effective implementation of Lean Six Sigma	Total Qual Manag Bus Excell	150+
2	Al-Balushi et al. [4]	2014	Readiness factors for Lean implementation in healthcare settings--a literature review	J Health Organ Manag	120+
3	Noori [9]	2015	Identifying critical issues in Lean implementation in hospitals	Hosp Top	100+
4	Kumar et al. [10]	2015	Conceptualisation of sustainable green Lean Six Sigma: an empirical analysis	Int J Business Excell	80+
5	Swarnakar et al. [7]	2023	Prioritization of critical success factors for sustainable Lean Six Sigma implementation in Indian healthcare organizations using best-worst method	TQM J	65+
6	Ali and Alolayyan [11]	2013	The impact of total quality management (TQM) on the hospital's performance: an empirical research	Int J Serv Oper Manag	60+
7	Spagnol et al. [12]	2013	Lean principles in healthcare: an overview of challenges and improvements	IFAC Proc Vol	55+
8	Sidek and Martins [13]	2017	Perceived critical success factors of electronic health record system implementation in a dental clinic context: an organisational management perspective	Int J Med Inform	50+
9	Rathi et al. [14]	2022	Lean Six Sigma in the healthcare sector: a systematic literature review	Mater Today Proc	40+
10	Naidoo and Fields [15]	2019	Critical success factors for the successful initiation of Lean in public hospitals in KwaZulu-Natal: a factor analysis and structural equation modelling study	Hum Resour Health	38+

An analysis of 262 papers revealed a fluctuating research focus on LSS-CSF healthcare studies. India-specific contributions ranged from 0 (0%) since 2013 to 1(1%) in 2022, showing negligible engagement. The analysis revealed significant geographical disparities, with developing countries like India contributing only 1 out of 262 (0.38%) publications, highlighting a critical research gap in emerging economies. More specifically, the year 2022 had only one India-centric paper, highlighting the inconsistent regional emphasis. This trend underscores sporadic research attention, reinforcing the identified gaps in the sustained scholarly focus on India’s healthcare LSS-CSF landscape (Table 3).

**Table 3:** Based on initial search results (n = 262), total number of papers and Indian specific papers retrieved.

Year	Total papers retrieved	India-specific papers	Percentage of Indian focus
2013	12	0	0%
2015	20	0	0%
2017	28	0	0%
2019	35	0	0%
2021	45	0	0%
2022	58	1	1.72%
2023	64	0	0%
Total	262	1	0.38%

The country-wise analysis of 262 publications revealed that the USA was the dominant contributor, followed by the UK and Canada. Australia ranked fourth, followed by China, South Africa, and Jordan. India trailed last with only 1 (0.38%) contribution. The findings highlighted significant geographical disparities, with Western nations leading research output, while developing countries such as India represented only 0.38% of the total publications (Table 4).

**Table 4:** Based on initial search results (n = 262), contribution of research by various countries.

Rank	Country	No. of publications	Percentage of contributions
1	USA	72	27.5%
2	UK	41	15.6%
3	Canada	23	8.7%
4	Australia	21	8.0%
5	China	18	6.9%
6	South Africa	10	3.8%
7	Jordan	8	3.0%
8	India	1	0.38%

The database analysis of 262 documents showed varying regional contributions, with Scopus database containing only one India-specific paper aligning with our research theme, and no other Indian healthcare-specific article was found in databases like Web of Science, followed by PubMed (Table 5).

**Table 5:** Results of various databases with the retrieved documents based on initial search results (n =262).

Database	Documents retrieved	Region-specific papers (India)
Scopus	200	1
Web of Science	12	0
PubMed	50	0

Analysis of CSFs from the identified studies was carefully studied, reviewed, and summarized under six main domains based on their frequency of occurrence (Table 6) [7-9,13,15,16]. Out of six domains, only four main domains were considered for further empirical investigations because only four domains have a frequency of occurrence ranging from high to moderate. Leadership and management as the most universal factors (8/8 studies). Communication was prominent (6/8), followed by training and expertise, and organizational readiness (5/8 and 3/8 each), whereas technology integration and employee retention, and culture were least cited (1/8).

**Table 6:** Summary of critical success factors (CSFs) identified in reviewed papers and their frequency of occurrence (n = 08).

CSF domains	Key authors referenced	Frequency
Leadership and management	Laureani and Antony [8]	High
Communication	Swarnakar et al. [7]; Naidoo and Fields [15]	High
Training and knowledge	Sidek and Martins [13]; Noori [9]	Moderate
Organizational readiness and structure	Al-Balushi et al. [4]; Sohal et al. [16]	Moderate
Technological integration	Sohal et al. [16]	Low
Employee retention and culture	Laureani and Antony [8]	Low

Table 7 presents a comparative analysis of eight healthcare studies that assessed the implementation of LSS and its associated CSFs for service quality improvement. The most consistently addressed components across studies include leadership and management, communication, training and staff development, and knowledge and learning, indicating their foundational importance in successful LSS execution. Leadership commitment appeared in most studies, although a few, such as those by Noori [9] and Naidoo and Fields [15], did not explicitly emphasize the involvement of senior management. Communication and training are universally acknowledged and underscore their critical role in enhancing staff capabilities. Organizational culture and social or environmental considerations were moderately represented, reflecting the growing attention paid to human and sustainability dimensions, albeit with inconsistency. Process and data management, particularly performance monitoring, were also a common focus, although aspects such as data management and the integration of lean tools were addressed less consistently. Notably, employee retention, technology, and financial governance are the least explored domains, suggesting that these areas remain underrepresented in the global healthcare CSFs impacting LSS literature. Overall, while there was a strong alignment of core CSFs, the variation in addressing other factors highlighted gaps in implementation strategies and suggested opportunities for more comprehensive and balanced approaches in future research.

**Table 7:** Details of studies assessing components of Lean Six Sigma (LSS) and critical success factors (CSFs) based on shortlisted qualitative review articles (n = 08).

Identified critical success factors in LSS implementation	Author and year							
	Aljazzazen and Schmuck (2022) [17]	Sohal et al. (2022) [16]	Swarnakar et al. (2021) [7]	Naidoo and Fields (2019) [15]	Laureani and Antony (2018) [8]	Sidek and Martins (2017) [13]	Noori (2015) [9]	Al-Balushi et al. (2014) [4]
Economic and effective managerial culture	No	No	Yes	No	Yes	No	No	No
Leadership and management	Yes	Yes	No	No	Yes	No	Yes	Yes
Data management	Yes	No	No	No	No	Yes	No	No
Good management system	Yes	No	No	No	No	No	Yes	No
Commitment of senior management	Yes	Yes	Yes	No	Yes	No	No	No
Talent recognition	Yes	No	No	No	Yes	No	No	No
Strategic leadership and organizational attitude	Yes	Yes	No	Yes	Yes	No	Yes	No
Technological attributes	Yes	No	Yes	No	No	No	No	No
Integration of lean elements, tools, and techniques	No	Yes	Yes	Yes	No	No	No	Yes
Skills and expertise	Yes	Yes	No	No	Yes	No	No	No
Social and environmental culture	No	No	Yes	No	Yes	No	No	No
High employee retention structure	Yes	No	No	No	No	No	No	No
Strategic orientation	No	No	No	No	No	No	Yes	No
Organizational culture	No	Yes	Yes	No	Yes	No	Yes	Yes
Open communication	Yes	Yes	Yes	No	No	Yes	Yes	Yes
Efficient organization structure	Yes	Yes	No	No	Yes	Yes	No	No
Requirements analysis and Performance monitoring	No	Yes	No	No	No	Yes	No	Yes
Managing finances effectively and team communication	Yes	Yes	No	No	Yes	No	No	No
Knowledge and learning	No	No	Yes	No	Yes	No	No	No
Training, staff training	No	No	Yes	No	Yes	Yes	No	Yes
Rewards	Yes	No	No	No	Yes	No	No	Yes
Stability and sustainability in operational process	No	Yes	No	Yes	No	No	No	No

Discussion

The bibliometric analysis conducted in this study provides a comprehensive overview of the research landscape surrounding LSS and its CSFs in the healthcare sector to improve service quality, with the main goal, revealing significant geographical disparities and highlighting the underrepresentation of developing countries, for instance, India's minimal presence in the literature. This global analysis identified a critical research gap: while LSS methodology has been extensively studied in developed countries, its application and adaptation in developing countries remain largely unexplored. India, representing one of the world's largest healthcare systems, serves as a prime example of this research deficit. By analysing 262 publications from Scopus, PubMed, and Web of Science, this study illuminates key trends, thematic clusters, and significant gaps in the application of CSFs for the successful implementation of LSS in healthcare settings. These findings underscore the global prominence of LSS as a process improvement methodology and highlight its growing but inconsistent adoption in India, a developing country with unique healthcare challenges. A comprehensive analysis conducted by Rathi et al. [14] revealed that the adoption of LSS in general, in the healthcare sector, began no earlier than 2005. While extensive research has been conducted regarding LSS in healthcare globally, there remains a relative scarcity of studies focused on developing countries, such as India and Brazil.

The keyword co-occurrence analysis, visualized through VOS-viewer, revealed a robust research ecosystem centred on core LSS methodologies such as "Six Sigma," "Lean, and “CSFs.” These terms formed a dense central cluster, reflecting their foundational role in the existing literature. The surrounding clusters, including "healthcare," "process improvement," and "service quality," indicating the interdisciplinary applicability of the methodology across sectors. The prominence of healthcare-specific terms like "patient safety" and "efficiency" in peripheral clusters highlights LSS’s practical relevance in addressing critical healthcare challenges, such as reducing medical errors and optimizing resource use [1,4,18]. However, the limited co-occurrence of "India" (8 co-occurrences) in the keyword network suggests that Indian healthcare remains an underexplored domain compared to manufacturing or Western healthcare contexts [14,18].

We conducted our search from 2012 to 2024, and academic researchers have predominantly concentrated on enhancing the healthcare systems of developed countries. In early 2012, the emphasis transitioned towards developing nations, given that these countries require more effective healthcare frameworks [19]. Since the focus of this study was to examine the impact of identified CSFs on LSS implementation in India, the present timeframe was chosen. The co-authorship network further emphasized the collaborative nature of the research theme, with key contributors such as Swarnakar et al. [7] playing central roles in advancing the field. The clustering of authors in practical applications (e.g., case studies) and empirical validation (e.g., surveys) reflects a balanced research approach [19]. However, the sparse representation of theoretical development and rural healthcare settings highlights critical gaps in the literature. These gaps are particularly significant in India, where rural hospitals face unique challenges such as resource constraints and limited infrastructure [20]. The post-2012 surge in LSS research, both globally and in India, aligns with the increasing emphasis on quality management and operational efficiency across sectors, driven by rising patient expectations and economic pressure [4,18,21].

An analysis of CSFs in Indian healthcare studies revealed that training and expertise were universally cited, underscoring their critical role in equipping staff to implement LSS effectively [7,8,13]. Leadership commitment and communication were also prominent, appearing in 8/8 and 6/8 studies, respectively, suggesting that managerial support and a conducive cultural environment are pivotal for success [8,16,17,22]. However, the inconsistent emphasis on organizational structure, employee retention, technology integration, and financial governance (cited in only 3/8 and 1/8 studies) highlights a significant shortfall in addressing these structural factors [23]. In India’s heterogeneous healthcare landscape, where public hospitals often lack advanced technology and funding, these gaps can hinder LSS implementation [18,24]. A moderate focus on organizational culture and social considerations indicates a growing awareness of human-centric factors, but their inconsistent application suggests the need for more comprehensive strategies [25].

The country-wise analysis revealed that Western nations, particularly the USA, UK, and Canada, dominate LSS research output, contributing significantly more than developing countries such as India and South Africa. This disparity reflects the differences in research infrastructure, funding, and academic focus [1,3]. India’s contribution, while notable (approximately 0.38% of 262 publications), is inconsistent, with only the year 2022 showing one India-specific study. This fluctuation suggests a lack of sustained research momentum, potentially due to limited institutional support or awareness of LSS’s benefits in Indian healthcare [14,18].

Top-cited articles, such as those by Laureani & Antony [8] and Al-Balushi et al. [4], highlight the global impact of LSS research in healthcare. These studies emphasize the methodology’s ability to enhance efficiency and quality, reinforcing its relevance in addressing universal healthcare challenges. However, the lower citation counts for Indian-focused studies, such as Swarnakar et al. [7], suggest that Indian research has yet to achieve comparable global influence. This may be attributed to the nascent stage of LSS adoption in India and the need for more robust context-specific studies [21,26].

The bibliometric findings align with existing literature reviews, such as Aljazzazen and Schmuck [17], who noted similar gaps in LSS research within the service sectors, particularly in developing countries. The limited exploration of CSFs, such as technology and infrastructure in Indian studies, mirrors global trends, where these factors are often overshadowed by human-centric CSFs such as leadership, communication, and training [16,17]. However, India’s unique socioeconomic and cultural dynamics, such as resource constraints, diverse patient demographics, and varying healthcare delivery models, necessitate tailored LSS strategies [18]. For instance, public hospitals in India often operate under severe budgetary and infrastructural limitations, making technology adoption challenging [20,23]. Addressing these context-specific barriers requires integrating LSS with innovative, low-cost solutions such as digital tools for process monitoring or scalable training programs.

Visualization of keyword co-occurrences and co-authorship networks provides a clear rationale for further investigation into Indian healthcare. Despite the country’s growing healthcare demand, the sparse representation of India-specific research underscores the need for targeted research. The identified clusters also suggest that LSS research in healthcare is multidisciplinary and integrates operational, managerial, and clinical perspectives. However, the limited focus on rural healthcare and theoretical frameworks provides opportunities for future research to bridge these gaps.

Clinical implications

These findings have significant global implications, particularly for healthcare systems in developing countries where resource constraints make LSS implementation both challenging and potentially highly beneficial. CSFs are those factors essential for the success of any program or technique, in the sense that, if objectives associated with the factors are not achieved, the application of the technique will perhaps fail catastrophically. The CSFs identified can help LSS to focus on reducing waste and variation, which can directly improve patient outcomes by minimizing wait times, reducing medical errors, and enhancing resource utilization. Leadership commitment ensures sustained support for LSS initiatives and fosters a culture of continuous improvement. However, the underemphasis on technology integration and data-driven decision making suggests that clinical settings may struggle to adopt data-driven LSS tools without adequate investment. Hospitals adopting LSS methodology can expect improved operational efficiency, reduced costs, and enhanced patient satisfaction, particularly in resource-constrained settings.

Future recommendations

Future research should prioritize India-specific LSS studies, particularly in rural and public healthcare settings, to address context-specific challenges. Developing theoretical frameworks for LSS implementation in resource-limited environments could enhance its applicability. The integration of technology, such as low-cost digital platforms for data management, should be explored to overcome infrastructural barriers. Longitudinal studies that assess the long-term impact of CSFs on LSS on patient outcomes and financial sustainability are required. Collaborative research between Indian and global scholars could elevate the visibility and impact of Indian studies by addressing geographical disparities in research outputs. Finally, increasing awareness of CSFs and LSS’s benefits through targeted training programs and policy advocacy could drive the broader adoption of LSS in Indian healthcare.

Limitations

This study has several limitations. The reliance on Scopus, PubMed, and Web of Science may have excluded relevant studies from other databases or non-English publications, thereby potentially limiting the comprehensiveness of the datasets. The focus of the recent literature (up to April 2024) may have overlooked older foundational studies. Bibliometric analysis, while effective in identifying trends, cannot capture the nuanced qualitative insights of individual studies. Additionally, one India-specific study out of 262 studies restricted the generalizability of the findings within the Indian context. Finally, the exclusion of non-peer-reviewed works and conference abstracts may have omitted emerging trends or practical insights from the grey literature.

## Conclusions

The bibliometric analysis and qualitative review highlight the growing yet uneven application of CSFs and their impact on LSS in healthcare, particularly in India, where research remains limited compared with global trends, revealing significant geographical disparities. This study reveals a robust LSS research ecosystem centred on core methodologies, with healthcare applications emphasizing efficiency, quality, and patient safety. However, the sparse focus on India-specific contexts, especially in rural and public healthcare settings, underscores this critical research gap. Key CSFs, such as leadership, communication, training, and organizational culture, are vital for successful LSS implementation. However, the neglect of technology integration and employee retention in Indian studies points to barriers in resource-constrained environments. These findings call for targeted research to develop context-specific LSS strategies and integrate innovative solutions to enhance healthcare delivery. By addressing these geographical research gaps, future efforts can drive sustainable improvements in healthcare systems across developing countries, with India serving as a potential model for similar emerging economies.
